# Odontoid process metastasis of bronchial carcinoma as a rare cause for nonmechanical neck pain: a case report

**DOI:** 10.4076/1757-1626-2-8173

**Published:** 2009-06-10

**Authors:** Stefan Lakemeier, Christina Carolin Westhoff, Susanne Fuchs-Winkelmann, Markus Dietmar Schofer

**Affiliations:** 1 Department of Orthopaedics, University Hospital Giessen and MarburgLocation Marburg, Baldingerstrasse, 35033 MarburgGermany; 2 Institute of Pathology, University Hospital Giessen and MarburgLocation Marburg, Baldingerstrasse, 35033 MarburgGermany

## Abstract

**Introduction:**

About 10% of spinal metastases are found in the cervical level. Magnetic resonance imaging is the gold standard for early detection of spinal metastases. The decision whether cervical spine magnetic resonance imaging is requested or not must be made clinically, taking into consideration the patients' detailed pain history.

**Case presentation:**

The case of an 85-year-old patient with a long history of neck pain caused by known multi-level cervical spine degeneration is presented. As conservative treatment became ineffective, he was sent for surgery. Pain character had changed from mechanical to nonmechanical. Therefore, new cervical Magnetic resonance imaging was requested, showing unexpected odontoid process osteolysis. Unknown lung cancer with adrenal and pancreatic metastases was revealed by further investigations.

**Conclusion:**

Detailed pain characterization can already indicate the correct diagnosis. In case of new onset cervical neck pain, magnetic resonance imaging should be performed soon, if pain is characterized as nonmechanical.

## Introduction

The spine is the most frequent site of osseous metastasis. 5% to 10% of all cancer patients develop spinal metastasis during the course of their disease. It is known that in adult patients up to 60% of spinal metastasis are either caused by breast, lung or prostate cancer with a preponderance of males [1]. Spinal metastases may occur in all ages and increase with higher age of the patient [2]. Most symptomatic spinal metastases are found in the thoracic part of the spine (70%) followed by the lumbar (20%) and cervical region (10%) [3]. Stark et al. reported that in one of ten cases of newly diagnosed spinal metastasis cancer was not known before [4].

Our report highlights a case of spinal metastasis of unknown lung cancer located in the odontoid process as a rare cause for nonmechanical neck pain and illustrates the complex quest for the correct diagnosis with contradicting imaging results while taking into account the patient's detailed pain history. To our knowledge this is the first case of lung cancer metastasis in this location described in the literature.

## Case presentation

We report the case of an 85-year-old healthy Caucasian man suffering from severe neck pain and headache for years with aggravation and a change in pain character since 2 months. On presentation at our department, he described his symptoms as constant boring pain not being altered by activity or rest whereas increasing at night and in the early morning. Physical exam showed a painful and restricted cervical spine motion. No sensory and motor deficits in the upper limbs were noted. General physical examination and laboratory investigations were normal. Past medical history revealed a heart attack 28 years ago and a 40 pack-year history of cigarette smoking. No regular medications were taken. He presented recent X-ray pictures showing a multi-level degeneration of cervical spine and 7-months-old MRI images showing cervical disc protrusion C4 to C7 and neuroforaminal stenosis with compression of the C6 nerve root considered to be the reason for his pain. As conservative treatment was ineffective, performance of spondylodesis and spinal decompression C5 - C7 was advised.

A neurological examination and a closer look at the patient's detailed pain history revealed that the abovementioned complaints could not be completely explained by the spinal stenosis. Therefore, new MRI scan was requested. An osteolysis of the odontoid process highly suspicious for metastasis was seen. CT scan of the cervical spine confirmed no compromise of odontoid process stability so that a pathologic fracture of the vertebra with possible severe consequences for the patient was not expected. Complete tumor staging with abdominal and thoracic CT scans and skeleton scintigraphy was performed. A coin lesion suspicious of bronchial carcinoma in the right lung and further pancreatic and adrenal lesions consistent with metastases were discovered. Further bony metastases could not be revealed. The histological examination of the tissue obtained in bronchoscopy displayed dense infiltrates of a large cell undifferentiated carcinoma in the right upper pulmonary lobe. Following the decision of the interdisciplinary tumor board, the patient was treated with palliative radiochemotherapy. This alleviated the patient's neck pain and headache. He was discharged from hospital on his request and died 14 weeks later in a hospice.

## Discussion

Pain in the cervical spine can be divided into two entities: tumor-related, nonmechanical and mechanical neck pain [5,6]. Tumor-related pain is due to the osseous destruction caused by tumor infiltration stretching the periosteum of the vertebra. It is noticed as a constant, boring pain with common sleep interruption. Conduction of the pain is either unilateral or bilateral in shoulder or head regions [5]. Mechanical pain caused by structural alteration of the cervical spine occurs with movement of the spine. It is reproducible and frequently due to fracture or instability [6]. Table 1 gives a detailed overview of the different reasons and clinical features of mechanical and nonmechanical neck pain.

**Table 1.** Comparison of mechanical and nonmechanical neck pain [5,6]

	Mechanical neck pain	Nonmechanical neck pain
Pain character	• Unilateral or bilateral pain	• Unilateral or bilateral pain
	• Often accompanied by severe headches	• Progressive worsening
	• Pain disappears in rest	• No relation to activities
	• Maximum of pain rather in the evening	• Frequent sleep interruption
	• Slow worsening	• No alteration by rest or activity
	• Activity related	• Maximum of pain in the early morning
	• Reproducible	• Patients feel head heaviness and instability
		• Significant loss of cervical spine rotation
Reason for pain	• Structural alterations of the cervical spine	• Tumor invasion into the vertebra
	○ (Pathologic) fracture	• Vertebral destruction stretching the periosteum
	○ Instability	• May become mechanical pain after pathologic fracture
	○ Degenerative cervical spine changes	
	○ Cervical strain	
	○ Whiplash-trauma	
	○ Rheumatological disorders	
Pain onset	• Insidious onset with slow worsening	• Insidious onset
	• Sudden onset after appropriate trauma	• Sudden onset after minimal trauma
Age of patients	• Patients of all ages	• Elder patients often with “red flacs” in their anamnesis:
	• Degenerative cervical spine disease rather in elderly patients	○ Tumor
		○ Trauma
		○ Infection
		○ Vascular disease
Radiological diagnostic steps	• X-ray imaging 6-8 weeks after unsuccessful conservative treatment	• Complete neurological examination
	• CT scan or MRI only in cases of neurological dysfunction or fracture	• Both X-ray imaging and MRI
Neurologic dysfunction	• Can be accompanied by all kinds of neurological dysfunction	• Relatively rare (5 to 10%)
		• Can be accompanied by all kinds of neurological dysfunctions.

The most available imaging modality is conventional X-ray picture. However, in osteolytic lesions 30% to 50% osseous destruction is needed for detectable changes on images [3]. Radioisotope bone scan being able to reveal lesions with as little as 3% to 5% alteration of the bone is far more sensitive. Nonetheless, this method is relatively non-specific and easy differentiation between fracture, infection, spondylosis or tumor is often impossible [7]. The gold standard for evaluation of spinal metastasis is MRI being able to detect cord and nerve compression as well as changes in the bone marrow very sensitively [8]. MRI also has the advantage of imaging the whole spine as in 30% of all cases multiple, clinically unexpected metastases are found [8]. However, since MRI is much more expensive than conventional X-ray imaging, it is not advisable that all patients with recent onset neck pain undergo MRI.

Following clinical guidelines, our department protocols radiological imaging starting with conventional X-ray pictures in patients with recent complaints of neck pain without neurological deficit and uneventful past medical history for trauma and tumor after six weeks of conservative treatment [9]. If X-ray findings are normal, cervical MRI scan is usually requested after another six weeks of unsuccessful physiotherapeutic treatment.

However, taking into account the increasing probability for cancer patients to suffer from spinal metastasis in relation to their age [10], cervical spine MRI scan is performed earlier in case of tumor-suspicious past medical history. Since this case, we will consider sending elderly patients with recent onset neck pain of nonmechanical character with normal X-ray findings for MRI sooner.

In the literature, various treatment options for cervical spine metastases are described. Radiochemotherapy is mentioned as being more effective to pain control than single surgical treatment [11]. Even in neurological emergency with rapid progressive paralysis, surgical intervention should be limited. Radiochemotherapy should be considered instead because life expectancy of lung cancer patients with spinal metastases is short. However, no prospective study has been done to compare surgical and nonsurgical treatment of spinal metastases [12]. In our case we think that radiochemotherapy helped to improve the patient's quality of life for his remaining lifetime.

**Figure 1.**
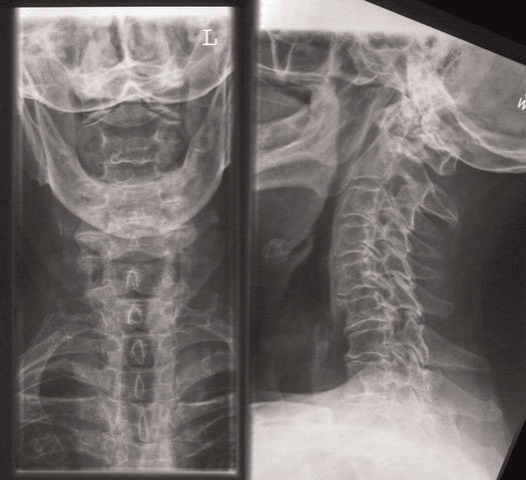
X-ray image of the cervical spine in two planes showing degenerative cervical spine disease with multi-level osteochondrosis. Odontoid process can not be seen.

**Figure 2.**
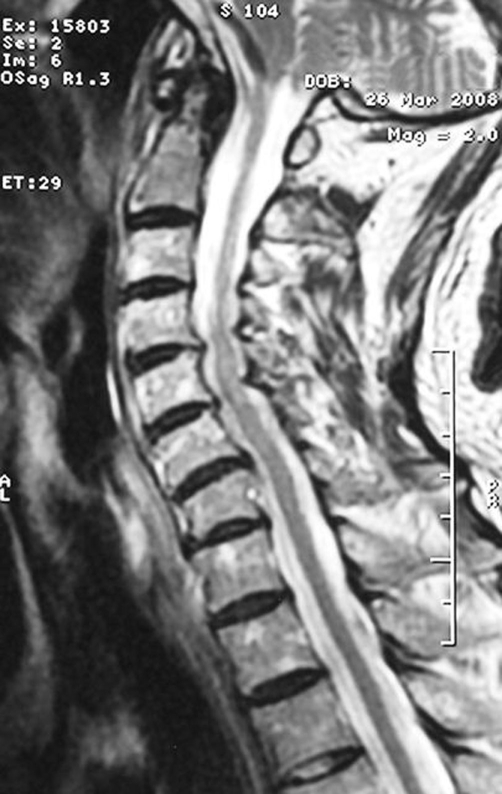
T2 weighted image of the cervical spine showing odontoid process osteolysis. Furthermore disc herniation of C4 to C7. No further osteolysis or fractures of the vertebra can be observed.

**Figure 3.**
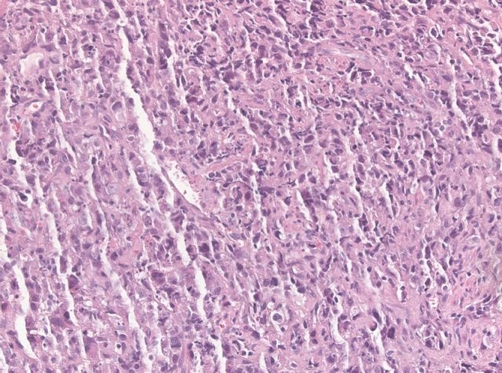
Histologic aspect of bronchoscopic biopsies from the right upper pulmonary lobe with medium-sized to large cells displaying moderate to extensive nuclear pleomorphism and extensive mechanical nuclear smearing and an increased number of apoptosis. Immunohistochemistry was negative except for CK7, prompting the diagnosis of large cell undifferentiated carcinoma (H&E).

## Conclusion

Spinal metastasis of unknown tumor can be a source of severe neck pain especially in elderly patients without cancer-related history. Pain character is different in metastatic and degenerative cervical spine disease. The clear description of the patient's complaints and the detailed history are essential for the definition of the correct diagnosis.

In cases of mechanic neck pain with no history of tumor or trauma, conventional X-ray imaging is a cheap and easily available imaging modality. MRI is the gold standard for early detection of bony metastases. The decision whether MRI is useful or not must be made considering the patients' detailed pain history.

We think that in single cases the clear clinical characterization of neck pain is more sensitive than sophisticated radiological examinations.

## Abbreviations

CK7: Cytokeratin 7
CT: Computed tomography
H&E: Hematosin and eosin stain
MRI: Magnetic resonance image
